# Effects of Supplemental Exogenous Emulsifier on Performance, Nutrient Metabolism, and Serum Lipid Profile in Broiler Chickens

**DOI:** 10.4061/2010/262604

**Published:** 2010-07-05

**Authors:** Amitava Roy, Sudipto Haldar, Souvik Mondal, Tapan Kumar Ghosh

**Affiliations:** Department of Animal Nutrition, Faculty of Veterinary Science and Animal Husbandry, West Bengal University of Animal & Fishery Sciences, 37 Kshudiram Bose Sarani, West Bengal, Kolkata 700037, India

## Abstract

The effects of an exogenous emulsifier, glyceryl polyethylene glycol ricinoleate, on performance and carcass traits of broiler chickens were assessed. The emulsifier was added to the diet at dose rates of 0 (control), 1 (E1) and 2 (E2) % of added fat (saturated palm oil). Live weight gain (*P* < .07) and feed conversion ratio (*P* < .05) in 39 days were higher in the E1 dietary group. Gain: ME intake and gain: protein intake during the grower phase improved quadratically (*P* < .05). Gross carcass traits were not affected. Body fat content and fat accretion increased (*P* < .05) and liver fat content decreased (*P* < .05) linearly with the level of emulsifier in diet. Fat excretion decreased (*P* < .001) leading to increased ileal fat digestibility (*P* < .06) in the E1 group (quadratic response). Metabolizable intake of N (*P* < .1) and fat (*P* < .05) increased quadratically due to supplementation of emulsifier in diet. Metabolism of trace elements and serum lipid profiles were not affected. The study revealed that supplementation of exogenous emulsifiers in diets containing moderate quantities of added vegetable fats may substantially improve broiler performance.

## 1. Introduction

Inefficient digestion and absorption of fat occurs in young chickens due to a low level of natural lipase production [1]. In chickens the activity and net duodenal secretion of lipase increases as the chick ages [2, 3]. A low rate of bile salt synthesis in young chicks further confounds the problem [4]. Dietary supplementation of bile salt reportedly improved fat utilization in chicks but the strategy may not be economically viable [1]. Therefore, cheaper emulsifying agents or detergents which transform a hydrophobic surface into a hydrophilic one have been used alternatively to increase fat digestibility in young chicks albeit with variable results [1]. Emulsifiers like soy lecithin promote incorporation of fatty acids into micelles and increase fat digestibility in chicks [5]. Synthetic emulsifiers like polyoxyethylene glycol mono- and dioleates have also been tried in pigs although the *in vivo* emulsification of fat by the synthetic emulsifiers was not found to be as effective as that obtained with bile salts [6]. Nevertheless, the exigency of using exogenous emulsifiers in broiler diets must be looked into because feeding of nutrient dense diets containing added fat is almost inevitable to exploit the full growth potential of the high yielding broiler strains.

In this experiment the effect of a synthetic emulsifier, glycerol poly ethylene glycol ricinoleate, supplemented in incremental dose levels, was ascertained in broiler chickens. Being amphiphilic in nature glycerol is essential for the uptake of the free fatty acids which are hardly soluble in bile salt micelles in the gut [7]. It was hypothesized that the said emulsifier would facilitate the process of emulsification *in vivo* to augment the digestibility of fat and nonfat nutrients including mineral elements. This should improve live weight gain, food conversion, nutrient metabolizability as well as mineral retention. The present investigation was carried out in a tropical climatic condition where dietary fat concentration hardly exceeds 3%-4% and one may argue the justification of an exogenous emulsifier with such a moderate quantity of added fat in diet. The aim of the study was to evaluate the dose response to the added emulsifier on performance and carcass traits, efficiency of nutrient utilization, and retention of trace elements in broiler chickens fed diets containing moderate quantity of added fat.

## 2. Materials and Methods

Cobb broiler chicks (*n* = 350, mean live weight 45.2 ± 0.89 g) were assigned to three dietary treatments for 39 days. The birds received feed within 12 hours of hatching. A pre-experimental slaughter (*n* = 20 birds) was performed on day 0. Each treatment group consisted of 9 replicates (*n* = 12 per replicate) and the replicates were placed in pens (1 m × 1.5 m) on litters composed of saw dust and rice husk. Temperature and lighting hours were maintained close to the recommended values [8] and the birds were vaccinated against Marek's disease, New Castle disease, and infectious bursal disease. The birds had *ad libitum* access to feed and drinking water. The starter (day 1 to 20) and the finisher (day 21 to 39) diets (Table 1) were formulated to meet or exceed the requirements [8]. Saturated palm oil (Bergafat HLL, Berg-Schmidt, GmBH and Co., Hamburg, Germany) was the source of supplemental fat. The fatty acid fraction of the supplemental fat was determined by gas chromatography as derivatized fatty acid methyl ester. All test diets were mashed and no antibiotic growth promoter was added. The cocktail enzyme (Enziver, RFCL, New Delhi, India) used in the dietary formulation did not contain lipase as a component.

The dietary treatments consisted of feeding the birds a basal diet without any added emulsifier (control), and the basal diet supplemented with glycerol poly ethylene glycol ricinoleate, E 484 (Volamel Extra, manufactured by Nukamel Inc., Hoogbuul, Olen, Belgium) at concentrations of 1% (E1) and 2% (E2) of the added fat (weight/weight). Therefore, each kg of the starter and finisher diets contained 350 mg and 280 mg of the emulsifier in the E1 and 700 mg and 560 mg of the emulsifier in the E2 dietary groups, respectively.

Live weight, feed consumption, and feed conversion ratio (FCR, feed intake g/live weight gain g) were measured replicate wise at every 10 days interval and the cumulative live weight gain and FCR in 39 days was calculated. Mortality, if any, was recorded and the aforementioned measurements were adjusted for mortality.

Apart from the pre-experimental slaughter, chicks were sacrificed on day 20 (3 birds per replicate) and day 39 (4 birds per replicate). The birds were killed after an overnight fast by exsanguination and the carcass were stored at –20°C for analyses. The hot carcass weight (after removal of the head, blood, neck, and hocks), eviscerated carcass weight and the yields of breast, frame, legs, wings, abdominal fat pad, and giblets (liver, heart, lungs, and gizzard) were determined on day 39. Breast weight included the breast fillet and the tenders (pectoralis major and minor) and the frame was defined as the eviscerated body frame with the head, neck, wings, legs, and breast removed. Moisture, protein, and fat in boneless meat samples of day 0, 20, and 39 were determined [9]. Samples for meat analysis were prepared by taking half of the longitudinally split carcass (including half of the abdominal fat pad) which were minced and mixed thoroughly until a subsample weighing approximately 500 g was achieved. Meat protein (N × 6.25) was determined in an automated Kjeldahl distillation apparatus (Kel Plus Calssic DX, Pelican Equipments, Chennai, India). For estimation of fat in meat and liver the moisture free dry samples were ground to pass through a 0.5 mm sieve and extracted with petroleum ether in an automated ether extract assembly (Socs Plus SCS 4, Pelican Equipments, Chennai, India) for 24 hours with the ether being changed at intervals of 8 hours. All the values concerned with the chemical composition of meat were expressed on fresh weight (FW) basis. Accretion of the aforesaid nutrients was determined between days 1 to 20, 21 to 39, and 1 to 39.

Blood was collected during the pre-experimental slaughter (*n* = 20) and subsequently on days 20 (*n* = 3 per replicate) and 39 (*n* = 4 per replicate) and the serum obtained thereof was stored at −20°C. Concentrations of glucose, total protein, albumin, cholesterol, HDL-cholesterol, and triacylglycerol in serum were determined in a semiautomatic blood biochemistry analyzer (Microlab 200, Merck, manufactured by Vital Scientific, Dieren, The Netherlands) using commercial kits (manufactured by Med-Source Ozone Biomedicals, Faridabad, India). All the assays were performed in duplicate and an intra assay variation of more than 5% was considered to be invalid and a fresh assay was performed.

A metabolism trial was conducted at the end of the feeding trial. The replicates, each containing 5 birds, were transferred to metabolism cages and placed there for 7 days including a collection period of 5 days. During the metabolism trial the amount of the food offered and that of the residues left was measured replicate wise accurately. The excreta were removed at every 2 hours interval and put in self-zipped polyethylene sachets and the total amount of excreta obtained in a 24 hours period was weighed. The excreta were manually mixed and a subsample measuring (1/20)th of the total excreta volume was kept daily in a hot air oven at 80°C for 16 hours to determine the dry matter (DM), organic matter (OM), and crude fat [8]. Another sub sample measuring (1/20)th of the total excreta volume was collected in broad mouth plastic containers for 5 days, pooled replicate wise and frozen at −20°C till analyzed for N and CP [8]. The diet was also analyzed for the above nutrients.

Metabolizability of DM and fat was determined with 3 birds per replicate at the end of the metabolism trial. The birds were selected randomly, slaughtered and the entire gastrointestinal tract was immediately exposed after splitting the carcass longitudinally. The ileum (from the Meckel's diverticulum to the ileocaecal junction) and the caecum were separated from the rest of the intestine and the contents present therein were separately collected by gently flushing into porcelain crucibles. Ileal digesta and caecal contents from birds within a replicate were pooled replicate wise, dried and stored at −20°C pending analysis of DM and fat. The contents recovered from the ileum and the caecum were considered to be undigested and the values were used for calculating the metabolizability of DM and crude fat.

Excretion and apparent absorption of copper (Cu), iron (Fe), manganese (Mn) and zinc (Zn) during the metabolism trial were determined. Approximately 2 g of oven dried feed and excreta sample was ignited in quartz crucible at 550°C for 4 hours. The cooled sample was treated with 3N nitric acid and boiled for 10 minutes under cover. The treated sample was filtered into a 50 mL volumetric flask and diluted up to the mark with deionized water (obtained from a Milipore Water Purification System, Elix 3, Mosheim, France). The concentration of Cu, Fe, Mn, and Zn was determined in an atomic absorption spectrophotometer (Perkin Elmer A Analyst 100, Wellesley, USA). To reduce interference due to presence of excess of Fe in samples a solution of ammonium chloride (20 mL/L) was added to the standard and the samples. Apparent retention coefficient was calculated as

(1) $$\frac{\left[\text{Trace}\;\text{element}*\text{feed}\;\text{intake}-\text{trace}\;\text{element}*\text{excreta}\right]}{\text{Trace}\;\text{element}*\text{feed}\;\text{intake}}.$$

All data were analyzed by multivariate analysis of variance in the general linear model (GLM) of the Statistical Package for Social Sciences [10]. The replicates were the experimental units and the concentration of supplemental emulsifier was the main effect. Data involving measurement at different time intervals (day) were analyzed by the repeated measure of GLM in which the effects of diet (dose of emulsifier), day (period of measurement) and diet-day interaction were determined. Orthogonal polynomial contrasts were applied to determine if the dose response was linear or quadratic. The data obtained prior to the start of the experiment were used as covariates. A probability value of *P* < .05 was described as statistically significant and that of *P* < .1 was described as a trend.

## 3. Results

Chemical composition of the starter and the finisher diets (Table 2) conformed to the requirements. The fatty acid fractions of the supplemental fat indicated that it contained 80% palmitic acid (C_16:0_) and 5% stearic acid (C_18:0_). The degree of unsaturation was not of much significance with the C1_18:1_ oleic acid constituting approximately 10% and C_18:2_ linoleic acid constituting 2% of the total lipid fraction of the fat source. The total fatty acid content was found to be 97%.

The birds remained healthy and consumed their daily meal allowances throughout the experiment. No mortality was recorded in the E2 dietary group during the grower phase. It was intriguing to note that 40% of the birds fed the control diet developed pasty vent within the first 4 days of the experiment. The figure stood at 15% and 10%, respectively, in the E1 and E2 dietary groups during this period. The birds received no veterinary treatment and the condition disappeared altogether in the latter two dietary groups by day 15 of the trial. However, in the control group more than 18% of the birds were still suffering from pasty vent condition (Table 3).

Live weight and live weight gain in 39 days was higher (*P* < .07) in the E1 dietary group. Cumulative feed consumption was similar across the dietary treatments (*P* > .1) although, during the grower phase the E1 group of birds consumed less food for each unit of live weight gain relative to the control and the E2 dietary groups (*P* < .05, quadratic effect). Numerically, overall feed: gain ratio in 39 days improved by approximately 5% in the E1 dietary group relative to that in the control group. Utilization efficiency of ME (gain: ME intake and gain: CP intake), which was not affected during the starter phase (*P* > .1), increased quadratically (*P* < .05) in the E1 dietary group during the grower phase compared to those in the control and the E2 dietary groups (Table 3).

Supplemental emulsifier did not affect (*P* > .1) the gross carcass traits (Table 4). Meat protein was found unchanged when measured on day 20 and day 39 (day effect *P* > .1) and supplemental emulsifier had little effect on this parameter (*P* > .1). Meat fat content increased with age (day effect *P* < .001) particularly in the E1 and E2 dietary groups (diet effect *P* < .001, linear effect, diet × day interaction *P* < .01). Consequently, fat accretion also increased linearly (*P* < .05) with the level of emulsifier in diet. Liver fat content decreased linearly (*P* < .05) with increasing level of emulsifier in diet and a diet-day interaction (*P* < .01) indicated towards an age-dependent effect of the emulsifier in this regard. Consequently, fat accretion in liver also tended to decrease linearly (*P* < .06) with the dose of the emulsifier in diet.

Supplemental emulsifier increased the metabolizability of DM and fat (Table 5). The ileal DM content was quadratically higher (*P* < .02) in the E1 dietary group and the caecal DM content was lower in the said group (*P* < .001, linear effect; *P* < .04, quadratic effect) compared to those in the control and the E2 dietary groups. Fat content in the ileum of the control and the treated groups was similar (*P* > .1). However, the caecal fat content decreased linearly with the dose of the emulsifier (*P* < .05) leading to an increased metabolizability of fat in the E1 dietary group (*P* < .1).

Added emulsifier had subtle effect (*P* > .1) on DM, OM, N and fat intake (Table 5). Excretion of N (*P* < .001, linear effect; *P* < .02 quadratic effect) and fat (*P* < .001, linear and quadratic effect) decreased with the dose of emulsifier. Excretion of DM and OM was not affected (*P* > 01) due to varying levels of added emulsifier and hence, intake of metabolizable DM and OM was similar across the treatment groups (*P* > .1). Metabolizable N intake tended to be higher (*P* < .1) and that of fat increased linearly (*P* < .05) with the dose of added emulsifier in diet (Table 5).

Intake, excretion and apparent absorption coefficient of the trace elements were mostly unaffected (*P* > .1) by supplementation of emulsifier to the diet (Table 6). Excretion of Zn increased linearly in the E1 and E2 dietary groups compared to that in the control group of birds (*P* < .05) although the apparent absorption coefficient of Zn was not affected. Apparent absorption coefficient of Cu was quite variable with the E1 group of birds showing higher Cu absorption than that in the control and the E2 dietary groups (*P* < .05).

Supplemental emulsifier had variable effects on serum metabolites (Table 7). Serum glucose increased linearly with the dose of supplemental in diet (*P* < .01). Serum glucose increased (day effect *P* < .001) as age of the birds advanced. Total protein and albumin concentrations were similar (*P* > .1) across the treatments. Total cholesterol and LDL cholesterol decreased linearly with the level of emulsifier in diet (*P* < .05) on day 20 although the difference disappeared on day 39 (*P* > .1). However, HDL cholesterol fraction remained unaffected (*P* > .1) by the level of emulsifier in diet. The HDL : LDL ratio widened (*P* < .05, linear effect) in the E1 and E2 groups day 20 although on day 39 the ratio was similar across the dietary treatments (*P* > .1). Added emulsifier exerted non significant effect in serum tryacylglycerol concentration (*P* > .1). It was observed further that irrespective of the level of emulsifier in diet serum tryacylglycerol concentration decreased (*P* < .001) with age.

## 4. Discussion

Fat content in the starter E1 and E2 diets was 1.6% and 3.9% higher than that in the basal diet. During the finisher phase fat content was 5.9% and 6.6% higher in the E1 and E2 diets, respectively, than that in the basal diet. Addition of the emulsifier, which was nothing but fat *per se *(ricinus oil ester) added to the crude fat content of the E1 E2 diets. Dietary concentration of trace elements exceeded the requirement [8] because prior to adding the mineral premix, concentration of the trace elements in dietary ingredients was not considered and so no adjustment was made in this regard.

Supplementation of emulsifier in incremental dose levels was expected to enhance utilization efficiency of dietary fat [7, 11] and improve live weight gain and feed conversion in broiler chickens. Conforming to the postulation there was 5% increment in live weight in the E1 dietary group relative to the control group of birds. Additionally, the birds supplemented with emulsifier markedly recovered from pasty vent condition perhaps due to better utilization of dietary fat. Improved utilization efficiency of ME and CP in the emulsifier supplemented birds during the grower phase, indicated positive effects of the emulsifier on digestion and absorption of fat as well as other nutrients. However, the quadratic dose responses suggested that with moderate quantities of fat added to diet, little benefit could be obtained if the concentration of supplemental emulsifier exceeds 1% of the added fat. The need of an emulsifier in broiler diet is more during the starter phase [12] because lipase activity in chickens reaches the peak only between 40 and 56 days of age [13]. Moreover, synthesis and recirculation of bile salts remain at a much lower plane in young chickens [14–16] and bile salts added to diets reportedly improved fat absorption in young chickens [5] and augmented productive performance [6, 7, 11, 17–19]. The results of the present investigation indicated the benefits of dietary supplementation of emulsifiers in grower phase also thus adding to the current status of knowledge.

It appeared from the liver fat content that supplementation of emulsifier probably facilitated shunting of fats more towards body depot and reduced fat deposition in liver [19]. As a result, fat accretion in whole body increased by approximately 50% in the emulsifier fed birds over that in the control group.

Subtle change in meat protein occurred despite greater utilization efficiency of CP particularly in the E1 group. This discrepancy could not be explained. However, CP utilization efficiency improved during the grower phase only and perhaps there was little room available for the muscle protein content to change during the entire course of the experiment.

Positive effect of emulsifiers on digestibility of nutrients is documented in pigs [7, 11, 19]. Supplemental emulsifier in the present study increased intake of metabolizable N and fat. Metabolizable N intake increased by 60.5% and 42.1%, respectively, in the E1 and E2 dietary groups over that in the control group of birds. Metabolizable fat intake also improved by approximately 17% and 14%, respectively, in the E1 and E2 dietary groups relative to the control group.

Supplementation of emulsifier improved metabolizability of DM and fat. However, the quadratic dose response confounds the justification of increasing the dose of the emulsifier beyond 1% of the added fat and warrants further research. The results corroborated an earlier work with pigs [20]. Lecithin reportedly depressed free fatty acid absorption from rat jejunum probably by increasing size of bile salt micelles which defuse more slowly through the luminal water interface and retarded delivery of free fatty acids to the absorptive cell surface [21]. Another possibility is that persistence of lecithin-bile salt micelles at the absorptive cell surface may alter free fatty acid partitioning. Free fatty acid absorption could be reduced if they prefer the aqueous environment of the mixed micelles rather than the lipid membrane of the absorptive cell surface [21]. Compared to lecithin, glyceryl polyethylene glycol ricinoleate is more hydrophilic and dissolves the free fatty acids which are hardly soluble in bile salt micelle alone [7] and thereby increases the digestibility of saturated fatty acids. It is possible that the higher concentration of the supplemental emulsifier in the E2 dietary group caused a greater number of fatty acid molecules to be released in vivo which were not absorbed efficiently since an excess of free fatty acids in gut lumen may interfere with the process of micelle formation [7, 22, 23]. The ileal fat content would perhaps support this hypothesis. The higher fat content in the cecum of the experimental birds compared to that in the ileum might be due to concentration of the cecal contents that occurred as a result of reabsorption of water from the cecal contents.

To our knowledge effect of an emulsifier on metabolism of trace elements has not been studied yet. Emulsifiers reportedly improved absorption of calcium and phosphorus [7, 11] although contrasting results are also available [17]. Nevertheless, the present experiment did not reveal any significant effect of supplemental emulsifier on metabolism of the trace elements. The variation observed with regard to Cu absorption could not be explained and may be ignored as an aberration.

Serum concentration of glucose indicated that more of the available glucose was utilized for growth and production in the E1 dietary group compared to that in the control and the E2 groups of birds. Serum lipid fractions seemed to be more vulnerable to the effects of supplemental emulsifier and it was reported that in pig serum tryacylglycerol decreased despite an enhancement in fat digestibility when lecithin was supplemented [11]. It was hypothesized that lecithin caused the chylomicrons to be cleared off from the blood at a faster rate or retarded their release into blood at a slower pace, thus lowering circulatory tryacylglycerol concentration. By augmenting the process of emulsification, lecithin increases the coat (phospholipids): core (tryacylglycerol) ratio of the lipoprotein fraction of chylomicrons and stabilizes the lipoprotein particles in the aqueous environment of the blood stream and decreases the release of the free fatty acids and cholesterol in blood [11]. However, insufficiency of knowledge regarding the effect of glyceryl polyethylene glycol ricinoleate on postabsorptive absorption of lipid and circulatory lipid profile precludes a definite conclusion about the exact mechanism that lowered the serum cholesterol concentration in the emulsifier supplemented birds.

The effect of emulsifier on HDL : LDL fraction of serum lipid in live stock is not ample. In pigs, LDL cholesterol may decrease when lecithin was fed as an emulsifier whereas lysolecithin raised circulatory LDL cholesterol [11]. However, similar reports with regards to synthetic emulsifiers are not available. However, the present investigation indicated that glyceryl polyethylene glycol ricinoleate may also reduce the cholesterol in serum of chickens. Liver fat concentration in the experimental groups was also suggestive of an efficient and rapid removal rate of lipids from the liver. However, fractionation of liver lipid content and determination of cholesterol metabolites in liver is warranted to reach at a confirmatory conclusion in this regard.

In conclusion, glyceryl polyethylene glycol ricinoleate was found to be an effective emulsifier for broiler chickens when added at concentrations above 1% of the added fat. At this concentration the emulsifier increased the live weight by approximately 5% and significantly improved feed conversion efficiency although the effects on carcass traits seem not to be very pronounced. There was certain effect on fat utilization which was evidenced from improvements in total tract apparent digestibility of fat and overall fat metabolizability which could be explored as a tool for enhancing fat utilization in high-yielding chicken fed greater quantity of added fat through diets. Overall, glyceryl polyethylene glycol ricinoleate may be considered as an inevitable feed additive component in the dietary regimen of high-yielding broiler chickens for augmenting nutrient utilization and food conversion in broilers.

## Figures and Tables

**Table 1:** Ingredients and chemical composition of the basal diet (g/kg as fed, unless stated other wise)^a,b^.

Ingredients	Starter	Grower	Chemical composition (calculated)	Starter	Grower
Maize	517.8	631.6	ME MJ	12.6	13.0
Soybean meal	412.0	314.0	Crude protein	232	195
Palm oil	35.0	28.0	Lysine	13.4	11.1
Limestone powder	9.0	7.0	Methionine	4.52	3.79
Di-calcium phosphate	12.6	6.5	Calcium	8.6	6.4
Sodium-bi-carbonate	1.1	1.1	Available phosphorus	4.3	3.2
Common salt	2.0	2.0	Sodium	2	1.5
Choline chloride (60%)	0.7	—	Chlorine mg	1850	1250
Lysine sulfate	1.4	1.6	Total digestible amino acids		
DL-methionine	2.3	1.9	Lysine	11.5	9.5
Threonine	0.3	0.5	Methionine	4.3	3.6
Mycotoxin binder	1.0	1.0	Methionine + cysteine	8.3	7.1
Ascorbic acid	0.1	0.1	Tryptophan	1.8	1.6
Organic acid blend^c^	1.0	1.0	Threonine	7.1	6.1
Cocidiostat	0.5	0.5	Arginine	12.1	10.3
Cocktail enzyme^d^	0.2	0.2	Isoleucine	7.5	6.4
Vitamin premix	2.0	2.0	Valine	8.9	7.5
Trace element premix^e^	1.0	1.0			

^a^The starter and the finisher diets were fed from days 1 to 20 and 21–39, respectively.

^b^The basal starter and finisher diets were supplemented with a nutritional emulsifier (glyceryl poly ethylene glycol ricinoleate, E 484, Volamel manufactured by Nukamel Inc., Hoogbuul, Olen, Belgium) at the dose rates of 0%, 1%, and 2% of the added fat (weight/weight).

^c^Contains (per kg) ortho-phosphoric acid (400 g), formic acid (150 g), propionic acid, and calcium propionate (30 g) mixed with a carrier.

^d^Enziver, manufactured by RFCL, New Delhi, India (contains protease, pectinase, cellulose, phytase, xylanase, amylase and ß-glucanase but no lipase).

^e^Contained (per kg) manganese 90 g, zinc 80 g, iron 90 g, copper 15 g (all as sulfate salt), iodine (as potassium iodide) 2 g, selenium (as sodium selenite) 0.3 g.

**Table 2:** Chemical composition of the experimental diets (analyzed values).

Attribute	Growth phase	Diet^a,b^		
		Control	E1	E2
Nutrients g/kg				
Moisture	Starter	173.1	176.3	172.8
	Grower	175.5	176.1	173.4
Organic matter	Starter	907	917.1	90.4
	Grower	915.6	917.7	918.3
Crude protein	Starter	229.3	230.1	230.4
	Grower	193.3	194.5	195.3
Crude fat	Starter	46.1	46.5	47.2
	Grower	53.9	56.8	56.9
Crude fiber	Starter	36.1	36.5	36.8
	Grower	34.2	33.9	34.1

Trace elements mg/kg diet dry matter				
Copper	Starter	14.7	14.2	14.5
	Grower	19.7	21.5	20.6
Iron	Starter	1031	1024	1016
	Grower	1604	1547	1502
Manganese	Starter	141.9	146.1	145.2
	Grower	153.8	150.8	150.9
Zinc	Starter	118.5	124.9	120.1
	Grower	111.2	107.3	110.8

^a^Analyzed on dry matter basis except moisture and organic matter; ^b^Supplemented with emulsifier at the dose rates of 0 (control), 1 (E1) and 2 (E2) percent of added fat (weight/weight basis).

**Table 3:** Live performance of broilers supplemented with exogenous emulsifier in graded doses^a^.

Response variables	Day	Dietary treatment			SE	Diet effect (contrast *P*)	
		Control	E1	E2		Linear	Quadratic
Performance traits							
Mortality (*n*)	1–20	2	2	1			
	21–39	1	1	Nil			
Pasty vent (%)	1–10	40	15	10			
	11–20	18.1	Nil	Nil			
	21–39	Nil	Nil	Nil			
Live weight g	1	51.2	50.8	51.4			
	10	264	268	269	1.92	.26	.86
	20	787	801	794	5.59	.63	.37
	30	1392	1438	1387	18.8	.92	.24
	39	2035	2136	2055	29.5	.76	.07
Live weight gain g	20	736	750	742	5.6	.64	.07
	39	1983	2085	2004	28.8	.77	.16
Food : gain	1–20	1.51	1.50	1.51	0.04	.99	.93
	21–39	2.08	1.93	2.07	0.03	.96	.05
	1–39	1.86	1.77	1.86	0.02	.31	.13
Gain : ME intake	1–20	53.2	53.2	52.8	1.34	.9	.94
	21–39	35.5	38.3	35.7	0.69	.94	.05
	1–39	40.5	42.6	40.5	0.58	.98	.11
Gain : CP intake	1–20	2.93	2.92	2.89	0.073	.89	.96
	21–39	2.49	2.67	2.48	0.043	.93	.05
	1–39	2.64	2.76	2.62	0.037	.82	.06

^a^Each treatment group consisted of 9 replicates (1–20 days, *n* = 12 per replicate; 21–39 days, *n* = 9 per replicate).

**Table 4:** Gross carcass traits and chemical composition of meat and nutrient accretion in broilers supplemented with exogenous emulsifier in graded doses (means of 3 birds/replicate on day 20 and 4 birds/replicate on day 39).

Response variables	Day	Dietary treatment			SE	Diet effect (contrast *P*)	
		Control	E1	E2		Linear	Quadratic
Gross carcass traits g							

Eviscerated carcass	39	1069	1137	1056	30.5	.87	.26
Breast	39	386	418	368	12.9	.57	.16
Legs	39	353	375	351	13.4	.97	.43
Abdominal fat	20	4.9	4.1	6.1	0.12	.39	.41
	39	19.2	20.2	16.9	0.89	.81	.88

Chemical composition of meat and liver g/kg fresh weight							

- Meat							
Protein	20	204	198	203	1.7	.77	.16
	39	200	196	208	2.9	.29	.21
Fat^a^	20	13.5	17.2	30.7	0.73	.0001	.007
	39	20.9	29.7	30.9	1.61	.02	.28
- Liver							
Fat^b^	20	32.4	28.3	24.1	0.21	.01	.12
	39	34.3	31.7	29.2	0.71	.02	.39
Liver fat accretion mg/day (1–39 day)		39.8	33.5	23.1	3.16	.43	.06

Nutrient accretion g							

Protein	1–20	144	143	145	1.93	.95	.67
	21–39	247	259	267	7.26	.29	.89
Fat^c^	1–20	9.7	12.9	23.5	0.55	.0001	.007
	21–39	32.4	49.7	38.3	2.96	.43	.037

^a^Day-diet interaction *P* = .01; ^b^Day-diet interaction *P* = .002; ^c^Day-diet interaction *P* = .02.

**Table 5:** Nutrient concentration in ileum and cecum and metabolizability of nutrients in broilers supplemented with graded levels of nutritional emulsifier^a^.

Response variables		Dietary treatment			SE	Diet effect (contrast *P*)	
		Control	E1	E2		Linear	Quadratic
Nutrient concentration in ileum and cecum g/kg digesta							

Ileal DM		189.9	207.7	185.7	3.55	.64	.02
Cecal DM		181.7	166.9	180.7	4.77	.0001	.04
Ileal fat		26.4	25.8	28.7	0.97	.35	.41
Cecal fat		36.1	31.2	34.8	0.23	.045	.0001

Metabolizability of nutrients							

Intake g/day	DM	185	191	184	3.77	.9	.4
	OM	169.9	175.9	169.6	3.42	.98	.41
	N	5.31	6.01	5.53	0.28	.75	.32
	Fat	9.89	10.5	10.4	0.24	.41	.46
Excretion (% intake)	DM	28.7	24.9	28.9	1.15	.94	.12
	OM	6.13	5.31	6.16	0.24	.95	.12
	N	46.4	19.6	21.3	2.64	.001	.02
	Fat	11.8	3.16	4.00	0.6	.0001	.0001
Metabolizable intake g/day	DM	132.9	143.9	130.9	4.22	.86	.19
	OM	159.6	166.6	159.2	3.44	.96	.34
	N	3.09	4.96	4.39	0.29	.08	.06
	Fat	8.74	10.19	9.96	0.25	.05	.12

^a^ *n* = 9 per replicate (each replicate consisted of 5 birds during the metabolism trial).

**Table 6:** Intake (mg/kg diet DM), excretion (mg/kg excreta DM), and apparent retention coefficient of trace elements in broilers during the metabolism trial (day 41–45) supplemented with graded levels of emulsifier^a^.

Response variables	Dietary treatment			SE	Diet effect (contrast *P*)	
	Control	E1	E2		Linear
*Intake*						
Copper	14.1	14.3	13.9	0.16	.56	.45
Iron	1573	1570	1555	5.89	.21	.6
Manganese	147	148	140	3.26	.39	.47
Zinc	109	109	108	0.79	.64	.78

*Excretion*						
Copper	8.4	9.3	10.8	1.19	.43	.2
Iron	905.5	913.1	917.4	8.14	.56	.92
Manganese	106.3	102.4	100.6	1.55	.15	.76
Zinc	63.2	66.3	66.6	0.54	.02	.23

*Absorption*						
Copper	0.291	0.367	0.213	0.04	.02	.06
Iron	0.383	0.303	0.36	0.03	.78	.36
Manganese	0.207	0.18	0.207	0.05	.99	.8
Zinc	0.375	0.272	0.332	0.04	.64	.31

^a^ *n* = 9 per replicate (each replicate consisted of 5 birds during the metabolism trial).

**Table 7:** Serum metabolite profile in broilers supplemented with graded levels of nutritional emulsifier^a^.

Response variables	Day	Dietary treatment			S.E.	Diet effect (contrast *P*)	
		Control	E1	E2		Linear	Quadratic
Glucose mmol/L	20	4.36	4.18	4.71	0.11	.22	.16
	39	8.09	7.82	9.64	0.12	.01	.04
Total protein g/L	20	36.6	37.7	33.7	1.03	.3	.25
	39	43.8	41.0	45.2	0.91	.68	.26
Albumin g/L	20	15.3	15.4	15.7	0.56	.7	.9
	39	12.3	12.7	11.9	0.53	.6	.9
Cholesterol mmol/L	20	5.46	4.13	3.88	0.32	.04	.43
	39	4.62	5.09	5.01	0.16	.43	.52
HDL cholesterol mmol/L	20	0.862	0.849	0.905	0.05	.74	.55
	39	0.998	0.909	0.935	0.04	.75	.54
LDL cholesterol mmol/L	20	4.6	3.28	2.97	0.29	.04	.44
	39	3.61	4.18	4.08	0.16	.38	.46
HDL : LDL	20	0.209	0.258	0.332	0.028	.09	.04
	39	0.288	0.221	0.256	0.02	.52	.26
Tryacylglycerol mmol/L	20	1.35	1.43	1.06	0.08	.55	.59
	39	0.7	0.79	0.71	0.11	.98	.68

^a^Data represent pooled mean of 9 replicate in each treatment group.
